# An instrument to measure atrial fibrillation knowledge in Chinese patients: validation of the Jessa Atrial fibrillation Knowledge Questionnaire

**DOI:** 10.3389/fphar.2023.1148524

**Published:** 2023-06-13

**Authors:** Sijie Chang, Wenlin Xu, Shuyi Wu, Lien Desteghe, Feilong Zhang, Jinhua Zhang

**Affiliations:** ^1^ Department of Pharmacy, Fujian Maternity and Child Health Hospital College of Clinical Medicine for Obstetrics & Gynecology and Pediatrics, Fujian Medical University, Fuzhou, China; ^2^ Department of Cardiology, Antwerp University Hospital, Antwerp, Belgium; ^3^ Research Group Cardiovascular Diseases, University of Antwerp, Antwerp, Belgium; ^4^ Faculty of Medicine and Life Sciences, Hasselt University, Hasselt, Belgium; ^5^ Heart Centre Hasselt, Jessa Hospital, Hasselt, Belgium; ^6^ Department of Cardiology, Fujian Medical University Union Hospital, Fuzhou, China

**Keywords:** atrial fibrillation, anticoagulant, knowledge, bleeding, validation

## Abstract

**Background:** There is no validated tool to assess patients’ knowledge of oral anticoagulant therapy in atrial fibrillation in China.

**Methods:** Using a standard translation program, the Jessa Atrial fibrillation Knowledge Questionnaire (JAKQ) was translated into Chinese. The reliability of the JAKQ was assessed by internal consistency (Cronbach’s α coefficient), repeatability (test-retest reliability), and sensitivity tests. Effectiveness was assessed by hypothesizing that a lower JAKQ score was a risk factor for bleeding. A total of 447 patients with atrial fibrillation (AF) who were hospitalized between July 2019 and December 2021 were studied and followed up. Participants were followed up 1, 3, 6, and 12 months after enrollment. Bleeding during follow-up was recorded. Data were obtained from hospital databases and telephone follow-up.

**Result:** A total of 447 patients with AF completed JAKQ. The mean age of patients was 67.7 ± 10.2 years. The median JAKQ score was 31.3% (12.5–43.8). The Cronbach’s α coefficient of JAKQ was 0.616–0.637, and the test-retest reliability value was 0.902 (*p* < 0.001). Multivariate logistic regression showed that the higher knowledge level of AF was associated with secondary education or above, an income of more than 2000 yuan, and a history of AF of more than 1 year. Bleeding was associated with a lower JAKQ score, hypertension, and a history of bleeding. Non-bleeding patients on VKA had a better understanding of how often INR should be monitored and what to do if an OAC dose was missed.

**Conclusion:** The Chinese version of JAKQ shows good reliability and validity, indicating that it is a valuable tool for AF and oral anticoagulation (OAC) knowledge assessment. It can be used in clinical practice to guide educational activities and improve the effectiveness and safety of treatment. It was shown that Chinese patients with AF have insufficient knowledge about AF and OAC. Lower JAKQ scores are associated with bleeding, so targeted education is necessary. Targeted educational efforts should focus on patients recently diagnosed with AF and those with lower formal education and income.

## Introduction

Atrial fibrillation (AF) is a common arrhythmia with a worldwide prevalence of 2%–4%, and the prevalence of AF increases with age (Hindricks et al., 2021). The risk of thrombotic disease in patients with AF is five times higher than that of the general population if left untreated, and the death or disability rate of AF-related stroke is twice as high as that of the population without AF (Migdady et al., 2021). Anticoagulant therapy in patients with AF reduces the risk of stroke by approximately 64% (Hart et al., 2007). Therefore, assessment of stroke risk and effective anticoagulation treatment, with vitamin K antagonists (e.g., warfarin) or non-vitamin K antagonist oral anticoagulants (NOACs), in patients are key (Lip and Lane, 2015; Kirchhof et al., 2016; Maruhashi and Higashi, 2020). However, anticoagulant therapy in patients with AF also increases the risk of bleeding. Major bleeding in patients with AF was reported in the range of 1.4%–5.2% in subjects treated with NOACs, and about 2.6%–5.6% in those on warfarin (Martinez et al., 2018; Guimarães et al., 2020; Weir et al., 2021; Rutherford et al., 2022).

After a patient is diagnosed with AF, it is necessary to decide whether to initiate anticoagulant therapy based on the risk of thrombosis (Pop et al., 2021). Meanwhile, it is also important to assess the risk of anticoagulant-related bleeding and inform the patient of the possible risks, complications, and expected benefits. Enhanced knowledge of diseases and drug use has improved clinical outcomes of disease treatment (including AF), drug safety as well as patient adherence (Man-Son-Hing and Laupacis, 2003; Fuenzalida et al., 2017; Guo et al., 2017; Vinereanu et al., 2017; Undas et al., 2020). Meanwhile, the 2020 ESC Guidelines for the diagnosis and management of atrial fibrillation state that better patient knowledge can help strengthen self-management and joint decision-making, and patients should be educated more comprehensively (Hindricks et al., 2021). At present, several questionnaires have been published to assess the knowledge level of patients with AF. Most of these studies were directed to a specific group of AF patients, e.g., patients new on OAC therapy (Smith et al., 2010; Clarkesmith et al., 2013), and patients undergoing radio-frequency catheter ablation (Xu et al., 2010). Some questionnaires focused on OAC therapy only, such as Oral Anticoagulation Knowledge test (Zeolla et al., 2006) and Oral Anticoagulation Knowledge Tool (AKT) (Obamiro et al., 2016). And some of the studies used tools that are often not practical in daily routine (Desteghe et al., 2016). Compared to these tools, the Jessa Atrial fibrillation Knowledge Questionnaire (JAKQ) includes knowledge about AF and anticoagulation. It is a brief, complete, and validated AF-specific knowledge questionnaire that can be used in daily practice to evaluate patients’ insight into their condition. It was designed in 2016 by Desteghe et al. and validated in a Belgian population (Desteghe et al., 2016; Desteghe et al., 2019). Since then, it has been repeatedly validated in the Polish (Konieczyńska et al., 2018; Janion-Sadowska et al., 2019) and Turkey populations (Özlü and Bayramoğlu, 2021). Currently, there is no validated knowledge assessment tool in China to examine patients’ knowledge level about oral anticoagulant (OAC) therapy. Therefore, the purpose of this study was to translate and validate the JAKQ, and to evaluate knowledge about AF and OAC in patients with AF at multiple centers in China.

## Methods

### Study design and setting

The study was conducted in seven centers: Fujian Medical University Union Hospital, the Affiliated Fuzhou First Hospital of Fujian Medical University, Guizhou Provincial People’s Hospital, Xinyang Hospital Affiliated to Zhengzhou University, The Second Affiliated Hospital of Chongqing Medical University, the Affiliated Hospital of Jining Medical University and First Hospital of Shanxi Medical University. All of the seven hospitals are Grade III A hospitals. Grade III A hospitals are the highest level of hospitals classified according to the current “Hospital Grading Management Measures” in China. (Supplementary Figure S1).

This prospective cohort study aimed to evaluate if the JAKQ applies to the Chinese population and to assess the knowledge level among patients with AF at seven centers between July 2019 and December 2021. The research is in line with the Declaration of Helsinki, and all patients provided written informed consent. The study has been registered in China Clinical Trial Registration Center (registration number: ChiCTR1900024455).

The inclusion criteria were as follows: 1) Age≥18 years old; 2) Outpatients and inpatients diagnosed with AF (regardless of primary or secondary diagnosis); 3) NOAC or VKA anticoagulant therapy should last at least 1 month; 4) Agree to sign the informed consent form. The exclusion criteria were as follows: 1) Cognitive impairment; 2) Severe liver and kidney insufficiency; 3) Severe heart failure; 4) Having experienced severe bleeding complications (e.g., cerebral hemorrhage and gastrointestinal hemorrhage) within the past 3 months; 5) Patients who did not want to be followed up. The inclusion and exclusion criteria were determined by querying the patient’s electronic medical record, searching for relevant information, or checking for any related diseases.

### The instrument

The knowledge level of patients with AF was evaluated using the JAKQ. The original JAKQ has gone through a complete validation process: i.e., content verification, face validation, response process, construct validity, internal consistency (Cronbach’s α0.522–0.674), test-retest reliability, sensitivity test, and identification potential (Desteghe et al., 2016). The JAKQ consists of 3 dimensions and 16 questions: part 1 about atrial fibrillation knowledge (8 questions); part 2 about general principles concerning anticoagulant therapy (5 questions); and part 3 about VKA therapy or NOAC therapy (3 questions). Each question contains four choices, with one point being given for choosing the correct answer and zero points being given for choosing a wrong choice or selecting the “I do not know” option. The score on JAKQ is divided by the number of completed questions to get the percentage. The anticoagulant knowledge of patients with AF was determined according to the correct answer rate. If the patient’s JAKQ score exceeded 50%, it was considered a high JAKQ score, otherwise it was considered a low score.

**FIGURE 1:**
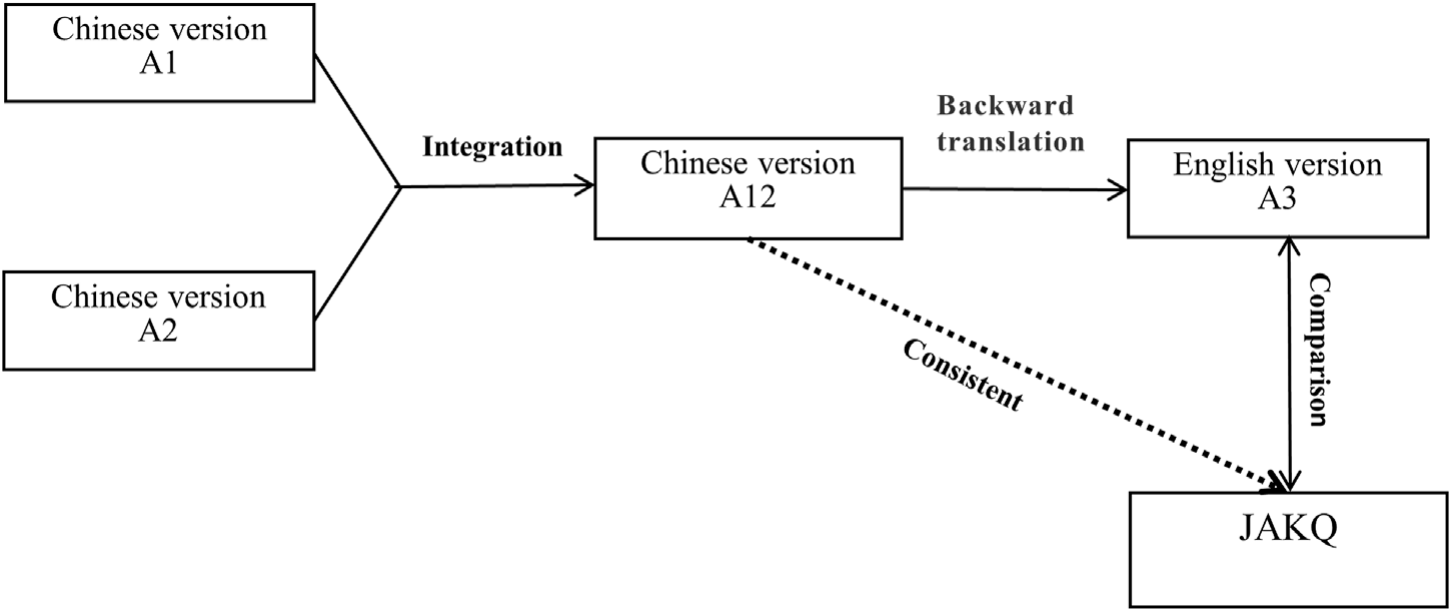
Translation process.

The WHO forward and backward translation method (World Health Organization, 2017) was used to translate the JAKQ. Two researchers proficient in Chinese and English translated the original JAKQ into Chinese versions A1 and A2, respectively, and another researcher combined the Chinese version A1 and A2 to form A12 and compared it with the original questionnaire. Then, a native English translator translated Chinese version A12 back into English version A3, and the English version A3 was compared with the original questionnaire. After repeated modification and backward translation, the Chinese version A12 was consistent with the original questionnaire, and a final version for testing was formed for a reliability and validity study (Figure 1).

### JAKQ validation

**FIGURE 2:**
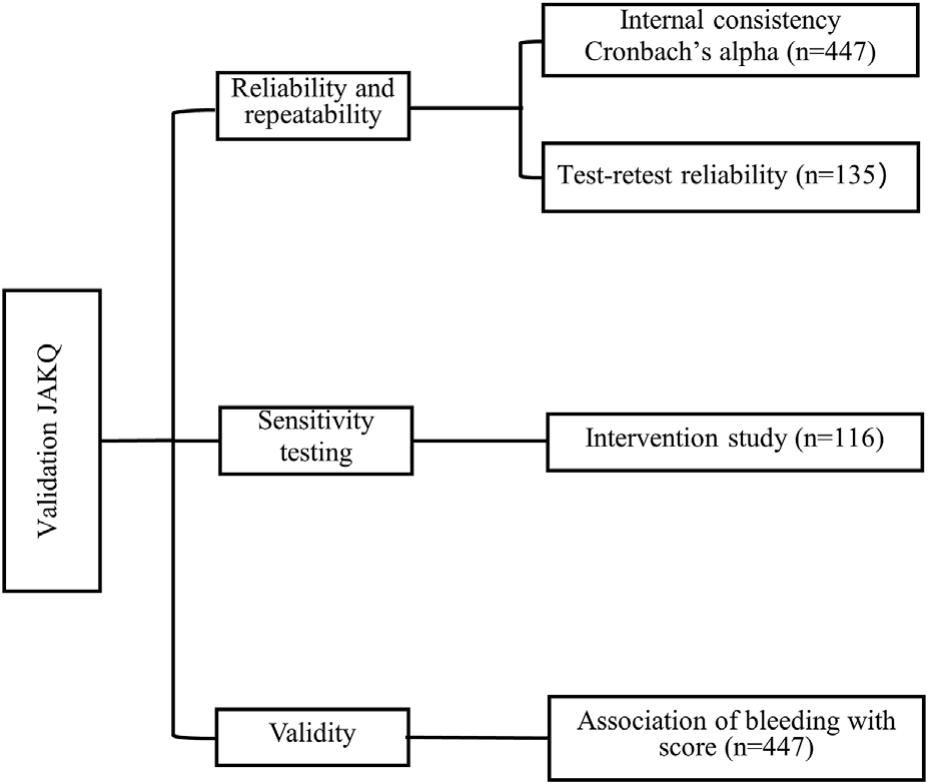
Overview of the different steps in the validation process of the Jessa Atrial fibrillation Knowledge Questionnaire.

The Chinese version of JAKQ validation included the analysis of reliability and validity. The internal consistency of JAKQ was determined by calculating Cronbach’s α coefficient. The reliability of JAKQ was determined by test-retest reliability to assess if the results on the JAKQ are consistent over time. A total of 135 patients (30%) completed the JAKQ with a retest after 1 month without receiving any educational intervention. Sensitivity analysis evaluated the results of targeted education in 116 patients. After all patients were enrolled, these two subgroups were selected by individuals who were not involved in this study through a random number table and agreed to by the patients. In the validity analysis, we hypothesized that lower JAKQ scores were associated with bleeding (Figure 2).

### Study procedure and targeted education

For patients who met the inclusion criteria, pharmacists administered the JAKQ to the patient at the end of the doctor’s clinic or before the patient was discharged. Then, under the guidance of a trained pharmacist, the patient independently completed the questionnaire. If the patient could not read, the researcher explained the questions and assisted the participants in completing the questionnaire. For patients who agreed to participate in retesting and education, the pharmacist reminded them to participate in the study at the hospital 1 and 3 months after enrollment. If the patient was unable to attend the hospital for some reason, the personal visit was replaced by a telephone follow-up.

Based on Desteghe et al.'s study(Desteghe et al., 2019), we designed targeted education, which was provided by a research team composed of pharmacists. After the patient completed the JAKQ, the pharmacist provided intensive education on the patient’s incorrect answers to the questionnaire. If the patient answered incorrectly, the pharmacist pointed out the correct answer to the patient and provided an explanation.

### Data collection

Data (demographic characteristics, education, income, duration of medication, time of diagnosis of AF) were collected from patients’ electronic medical records, and information on bleeding events was obtained through follow-up of patients or their relatives. Participants were followed up 1, 3, 6, and 12 months after enrollment. Major bleeding was defined according to the International Society for Thrombosis and Hemostasis (ISTH), as occurring in a critical organ (Intracranial, intraspinal, intraocular, retroperitoneal, intra-articular or intra-pericardial, intramuscular compartment syndrome) or as a drop in hemoglobin level of at least 2 g/dL or transfusion of at least 2 units of red blood cells. Minor bleeding events were defined as not meeting the criteria for major bleeding or clinically significant bleeding (Schulman and Kearon, 2005). CHA2DS2-VASc and HAS-BLED scores were the results of scoring based on the patient’s medical record display data.

### Statistical analysis

SPSS 25.0 (IBM, Armonk, USA) was used for statistical analysis. Kolmogorov-Smirnov test was used to examine whether the continuous variables conformed to the normal distribution, and Levene’s test was used to examine whether the two groups of variables had homogeneity of variance. Continuous variables were expressed by mean ± standard deviation (SD) or median and inter-quartile range (IQR). Categorical variables are reported as relative number component ratio (%) or rate (%). Spearman’s rho and Mann-Whitney U test were used to evaluate the test-retest reliability of JAKQ. The effect of targeted education was evaluated by the Mann-Whitney U test. The Chi-square test and *t*-test were used to compare demographic differences. Univariate binary logistic regression analysis was used to evaluate the correlation factors affecting questionnaire scores and bleeding. All variables with *p* < 0.1 in univariate analysis were used for multivariate logistic regression analysis. A *p*-value <0.05 was considered statistically significant.

## Results

### Patient characteristics

Of the 485 patients with AF who requested to participate, 38 patients were excluded because they did not meet the inclusion criteria (n = 13) or refused follow-up (n = 7), and 18 patients were lost to follow-up at the end of the one-year follow-up. A total of 447 patients with AF were included in the final analysis, half of which were females (45.9%). The majority of patients received NOACs (62.9%), namely, rivaroxaban 185 (65.8%) as well as dabigatran 96 (34.2%), while 37.1% of patients were treated with VKA. More than half of the patients (51.2%) had hypertension, and 340 patients (76.1%) had taken anticoagulants for less than 1 year (Table 1).

**TABLE 1:** Characteristics of the study population.

	All (n = 447)	Patients with bleeding (n = 133)	Patients without bleeding (n = 314)	*p*-Value
Age (years), mean ± SD	67.72 ± 10.24	65.69 ± 11.56	68.96 ± 9.20	0.072
Female, n (%)	205 (45.9%)	45 (33.8%)	160 (51.0%)	0.001
Weight (Kg), mean ± SD	63.37 ± 10.02	64.02 ± 11.00	62.97 ± 9.42	0.558
CHA2DS2-VASC, mean ± SD	3.41 ± 1.94	3.16 ± 1.85	3.57 ± 1.96	0.238
HASBLED, mean ± SD	1.91 ± 1.06	1.94 + 0.99	1.89 ± 1.10	0.793
Type of anticoagulant, n (%)				
VKA	166 (37.1%)	59 (44.4%)	107 (34.1%)	0.04
NOACs	281 (62.9%)	74 (55.6%)	207 (65.9%)	
Degree of education, n (%)				
Illiteracy	62 (13.9%)	6 (4.5%)	56 (17.8%)	<0.001
Primary education	149 (33.3%)	55 (41.4%)	94 (29.9%)	
Secondary education	173 (38.7%)	65 (48.9%)	108 (34.4%)	
Tertiary education	63 (14.1%)	7 (5.3%)	56 (17.8%)	
Time since AF diagnosis, n (%)				
<1 month	58 (13.0%)	8 (6.0%)	50 (15.9%)	<0.001
1 month −1 year	167 (37.4%)	74 (55.6%)	93 (29.6%)	
1 year −5 years	151 (33.8%)	40 (30.1%)	111 (35.4%)	
>5 years	71 (15.9%)	11 (8.3%)	60 (19.1%)	
Income, n (%)				
None	105 (23.5%)	26 (19.5%)	79 (25.2%)	<0.001
<2000yuan	64 (14.3%)	18 (13.5%)	46 (14.6%)	
2000–5000yuan	189 (42.3%)	79 (59.4%)	110 (35.0%)	
>5000yuan	89 (19.9%)	10 (7.5%)	79 (25.2%)	
Baseline disease, n (%)				
Hypertension	229 (51.2%)	101 (75.9%)	128 (40.8%)	<0.001
Diabetes	88 (19.7%)	24 (18.0%)	64 (20.4%)	0.570
Heart failure	84 (18.8%)	36 (27.1%)	48 (15.3%)	0.004
Stroke	49 (11.0%)	10 (7.5%)	39 (12.4%)	0.129
Drug combination, n (%)				
Antiplatelet agents	52 (11.6%)	15 (11.3%)	37 (11.8%)	0.879
Statins	172 (38.5%)	53 (39.8%)	119 (37.9%)	0.698
ACEI	108 (24.2%)	37 (27.8%)	71 (22.6%)	0.240
Amiodarone	90 (20.1%)	37 (27.8%)	53 (16.9%)	0.008
β-receptor blockers	83 (18.6%)	22 (16.5%)	61 (19.4%)	0.473
Time of taking anticoagulant, n (%)				
≤1 year	340 (76.1%)	126 (94.7%)	214 (68.2%)	<0.001
>1 year	107 (23.9%)	7 (5.3%)	100 (31.8%)	
History of bleeding, n (%)	51 (11.4%)	26 (19.5%)	25 (8.0%)	<0.001
History of thrombosis, n (%)	71 (15.9%)	36 (27.1%)	35 (11.1%)	<0.001

### JAKQ validation

JAKQ had an acceptable internal consistency. The Cronbach’s α coefficient for the Chinese version of JAKQ was 0.746, the Cronbach’s α coefficient of each dimension was 0.637 for atrial fibrillation related questions; 0.634 for the part about oral anticoagulation and the questions about VKA and 0.616 for the part about general anticoagulant knowledge in combination with the NOAC questions.

The demographic characteristics of the subgroups in the test-re-test and intervention studies are shown in Table 2. The demographic characteristics of the two subgroups were similar to those of the entire cohort. The Test-retest reliability analysis showed no significant difference between patients’ JAKQ scores at baseline and after 1 month if no additional education was provided (37.5% (12.5–50.0) vs. 37.5% (12.5–50.0), *p* = 0.674). This indicates that JAKQ has good reliability (rho = 0.902, *p* < 0.001).

**TABLE 2:** Characteristics of the subgroups in the test-re-test and intervention studies.

	All (n = 447)	Patients in the test-re-test (n = 135)	*p*-value^a^	Patients with education (n = 116)	*p*-value^a^
Age (years), mean ± SD	67.72 ± 10.24	67.06 ± 9.01	0.820	65.18 ± 9.16	0.428
Female, n (%)	205 (45.9%)	66 (48.89%)	0.537	46 (39.66%)	0.231
Weight (Kg), mean ± SD	63.37 ± 10.02	64.80 ± 9.74	0.260	66.41 ± 10.88	0.027
CHA2DS2-VASC, mean ± SD	3.41 ± 1.94	2.76 ± 1.54	0.059	2.51 ± 1.82	0.017
HASBLED, mean ± SD	1.91 ± 1.06	1.63 ± 0.93	0.136	1.41 ± 1.02	0.013
Type of anticoagulant, n (%)					
VKA	166 (37.1%)	57 (42.22%)	0.287	36 (31.30%)	0.222
NOACs	281 (62.9%)	78 (57.78%)		80 (68.97%)	
Degree of education, n (%)					
Illiteracy	62 (13.9%)	20 (14.81%)	0.552	19 (16.38%)	0.906
Primary education	149 (33.3%)	37 (27.41%)		36 (31.30%)	
Secondary education	173 (38.7%)	60 (44.44%)		45 (38.79%)	
Tertiary education	63 (14.1%)	18 (13.33%)		16 (13.79%)	
Time since AF diagnosis, n (%)					
<1 month	58 (13.0%)	15 (11.11%)	0.859	10 (8.62%)	0.647
1 month −1 year	167 (37.4%)	49 (36.30%)		46 (39.66%)	
1 year −5 years	151 (33.8%)	46 (34.07%)		41 (35.34%)	
>5 years	71 (15.9%)	25 (18.52%)		19 (16.38%)	
Income, n (%)					
None	105 (23.5%)	37 (27.41%)	0.149	28 (24.14%)	0.033
<2000yuan	64 (14.3%)	14 (10.37%)		14 (12.07%)	
2000–5000yuan	189 (42.3%)	66 (48.89%)		37 (31.90%)	
>5000yuan	89 (19.9%)	18 (13.33%)		37 (31.90%)	
Baseline disease, n (%)					
Hypertension	229 (51.2%)	79 (58.52%)	0.817	65 (56.03%)	0.722
Diabetes	88 (19.7%)	28 (20.74%)		19 (16.38%)	
Heart failure	84 (18.8%)	24 (17.78%)		19 (16.38%)	
Stroke	49 (11.0%)	13 (9.63%)		14 (12.07%)	
Drug combination, n (%)					
Antiplatelet agents	52 (11.6%)	16 (11.85%)	0.785	9 (7.76%)	0.361
Statins	172 (38.5%)	62 (45.93%)		47 (40.52%)	
ACEI	108 (24.2%)	30 (22.22%)		25 (21.55%)	
Amiodarone	90 (20.1%)	33 (24.44%)		33 (28.45%)	
β-receptor blockers	83 (18.6%)	32 (23.70%)		24 (20.69%)	
Time of taking anticoagulant, n (%)					
≤1 year	340 (76.1%)	103 (76.30%)	0.955	95 (81.90%)	0.182
>1 year	107 (23.9%)	32 (23.70%)		21 (18.10%)	
History of bleeding, n (%)	51 (11.4%)	26 (19.26%)	0.018	10 (8.62%)	0.389
History of thrombosis, n (%)	71 (15.9%)	27 (20.00%)	0.263	26 (22.41%)	0.097

^a^ Compared with all patients.

**FIGURE 3:**
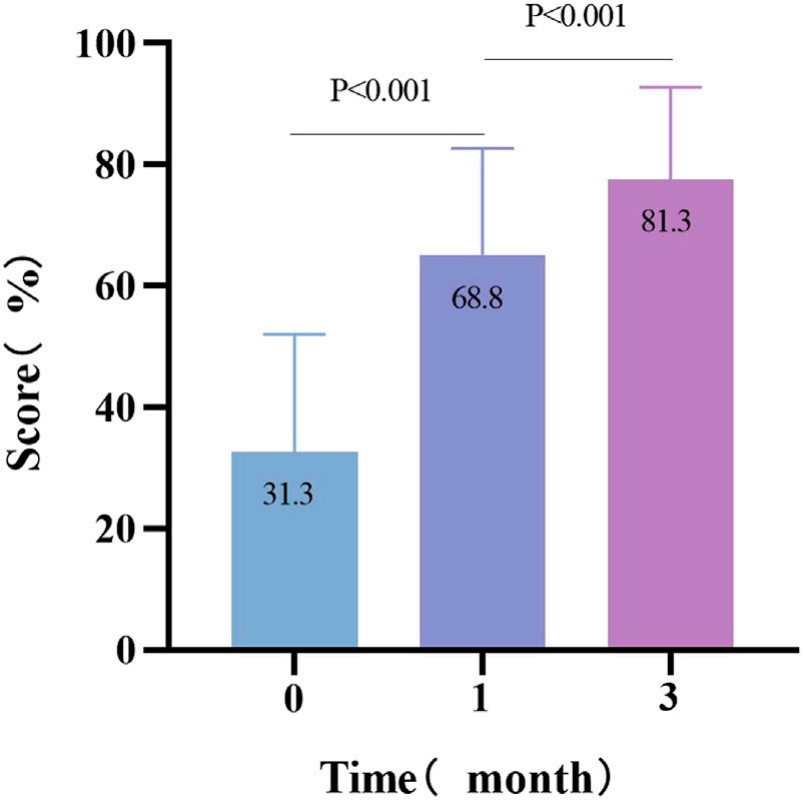
Effect of targeted education on the score of the Jessa AF Knowledge Questionnaire (JAKQ).

To test the sensitivity of the questionnaire, the effect of targeted education was assessed in the population. Compared with baseline JAKQ scores, patients scored significantly better about 1 month after they had received targeted education (31.1% (18.8–43.8) vs. 68.8% (50.0–75.0), *p* < 0.001). After 2 months of additional education, the learning effect was still significant (68.8% (50.0–75.0) vs. 81.3 (68.8–87.5), *p* < 0.001) (Figure 3).

### Knowledge of patients with AF

The scores of Chinese patients with AF were generally low (31.3% (12.5–43.8)), with a minimum score of 0 and no one getting the full score. Notably, only 5.1% of patients knew that painkillers based on paracetamol were the safest painkillers combined with OAC treatment. At the same time, 91.8% of patients on NOACs were not aware of the role of the OAC cards or were not aware of the existence of OAC cards at all. In addition, only 17.3% of patients knew they could detect AF by taking their pulse regularly. The majority of patients using NOACs (85.1%) did not know what to do when the OAC dose was missed, compared with patients using warfarin (52.9%). Interestingly, 71.1% of patients on warfarin were sufficiently aware of the importance of regular monitoring of INR (Table 3).

**TABLE 3:** Specific topics addressed in the Jessa AF Knowledge Questionnaire (JAKQ) with the percentage of correct responses among patients with or without bleeding.

	All (n = 447)	Patients with bleeding (n = 133)	Patients without bleeding (n = 314)	*p*-Value
Q1-1 AF is a condition where the heart beats irregularly and often faster than normal	216 (48.3%)	48 (44.4%)	168 (49.6%)	0.354
Q1-2 AF is not always accompanied by symptoms	177 (39.6%)	47 (43.5%)	130 (38.3%)	0.339
Q1-3 Patients can detect AF by taking their pulse regularly	77 (17.3%)	18 (16.6%)	60 (17.6%)	0.806
Q1-4 AF can cause blood clots which can lead to stroke	220 (49.2%)	49 (45.4%)	171 (50.4%)	0.359
Q1-5 Medication cannot prevent AF permanently, as the arrhythmia will increasingly occur with ageing, even when taking medication	108 (24.2%)	23 (21.3%)	85 (25.1%)	0.424
Q1-6 An AF patient should not go to the general practitioner or emergency room each time he/she feels AF	112 (25.1%)	51 (47.2%)	61 (18.0%)	<0.001
Q1-7 Being overweight exacerbates AF	120 (26.9%)	26 (24.3%)	94 (27.7%)	0.455
Q1-8 Blood thinners are often prescribed for patients with AF in order to prevent the development of blood clots in the heart, which can lead to stroke	208 (46.5%)	48 (44.4%)	160 (47.2%)	0.617
Q2-1 Patients with AF should always take their blood thinners, even if they do not feel AF	128 (28.6%)	29 (26.9%)	99 (29.2%)	0.638
Q2-2 Possible side effects of blood thinners are the occurrence of bleedings and longer bleeding times in case of injuries	142 (31.8%)	33 (30.7%)	109 (32.2%)	0.756
Q2-3 AF patients may only take painkillers based on paracetamol	23 (5.1%)	7 (6.3%)	16 (4.7%)	0.470
Q2-4 When AF patients regularly have minor nose bleeds (that spontaneously cease), they should contact the general practitioner or specialist, while continuing to take their blood thinners	109 (24.4%)	25 (23.3%)	84 (24.7%)	0.731
Q2-5 If an AF patient needs an operation, he/she should consult a doctor to discuss possible options	155 (34.6%)	39 (36.2%)	116 (34.1%)	0.719
Q3-1 AF patient taking VKA should have their blood thinning checked at least once a month	118 (71.1%)	30 (50.8%)	88 (82.2%)	<0.001
Q3-2 When AF patient taking VKA have forgotten to take their blood thinner, they should still take their forgotten pill immediately or at the next dose	78 (47.1%)	18 (30.5%)	60 (56.1%)	0.002
Q3-3 INR is a measure to check how thick or how thin the blood is	73 (43.9%)	21 (35.6%)	52 (48.6%)	0.106
Q4-1 For patients taking NOAC, it is important to take their blood thinner at the same time every day	123 (43.9%)	37 (50.0%)	86 (41.5%)	0.208
Q4-2 When AF patients taking NOAC have forgotten to take their blood thinner, they can still take that dose, unless the time till the next dose is less than the time after the missed dose	42 (14.9%)	13 (17.6%)	29 (14.0%)	0.461
Q4-3 The NOAC card should be shown to their general practitioner and specialist by AF patients	23 (8.2%)	2 (2.7%)	21 (10.1%)	0.045

### Factors influencing questionnaire score

**FIGURE 4:**
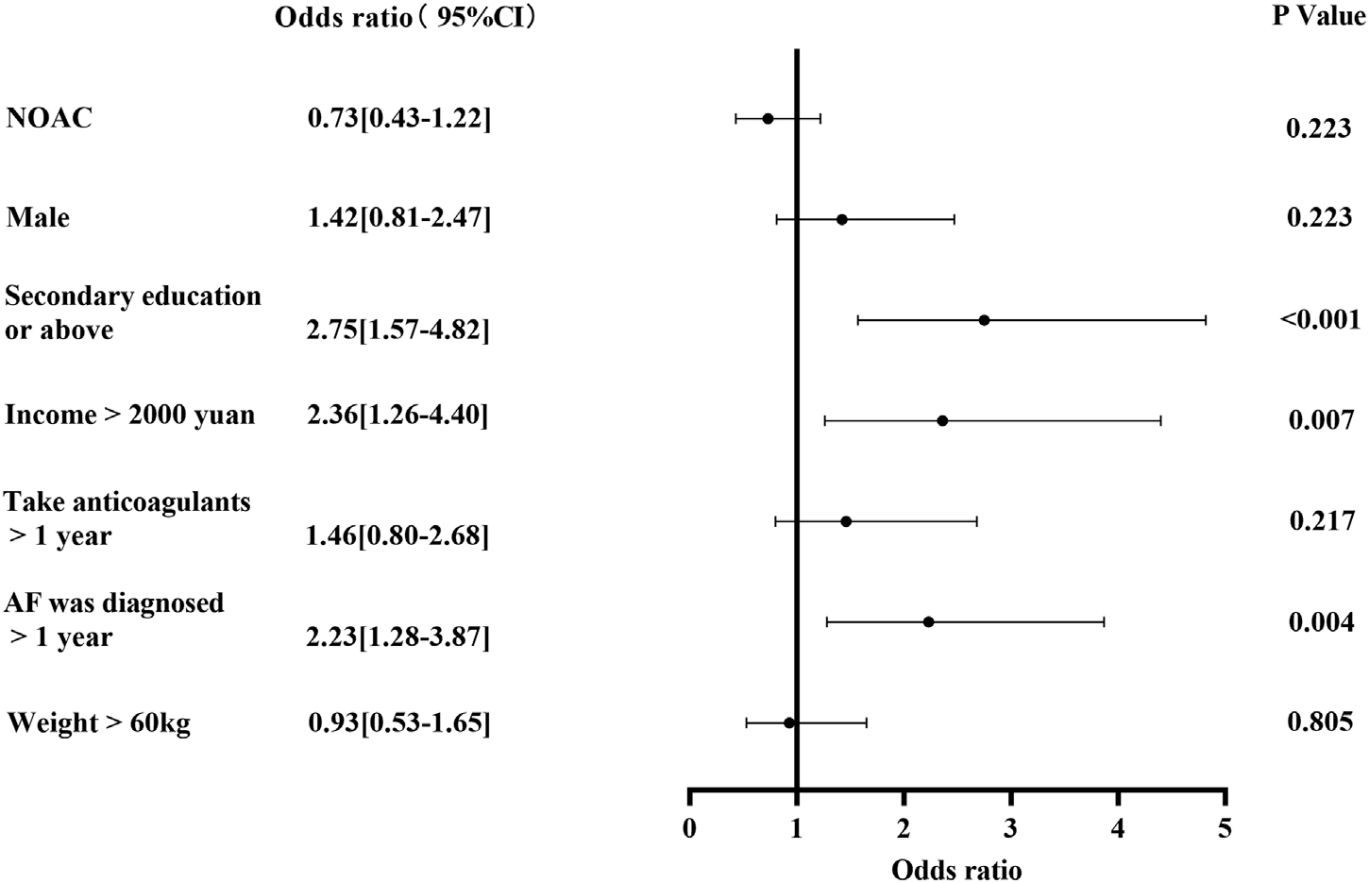
Factors associated with higher JAKQ score (A JAKQ score exceeding 50% was considered a high JAKQ score, otherwise it was considered a low score).

In the multivariate-adjusted logistic regression model, secondary education or above (OR 2.75; 95%CI, 1.57–4.82; *p* < 0.001), diagnosed with AF for more than 1 year (OR 2.23; 95%CI, 1.28–3.87; *p* = 0.004) and income more than 2000 yuan (OR 2.36; 95%CI, 1.26–4.40; *p* = 0.007) was significantly correlated with the higher knowledge level of AF (Figure 4).

### Knowledge and bleeding

Of note, patients with a bleeding event during 1 year follow-up, scored less on JAKQ than those without a bleeding event (25.0% (12.5–43.8) vs. 37.5% (25.0–50.0), *p* < 0.001). Non-bleeding patients on VKA had a better understanding of how often INR should be monitored (82.2% vs. 50.9%, *p* < 0.001) and what to do if an OAC dose was missed (56.1% vs. 30.5%, *p* = 0.002). Fewer patients with AF with bleeding knew the purpose of OAC cards accompanying NOACs compared to those without bleeding (10.1% vs. 2.7%, *p* = 0.045). Notably, patients with bleeding were more aware than those without bleeding (47.2% vs. 18.0%) that they should not go to the general practitioner each time when they feel AF (Table 3).

### The difference in patients with and without bleeding

Bleeding was observed in 133 patients (29.7%) and was more often reported by males than females (66.2% vs. 33.8%). Among the bleeding patients, 59 patients (44.4%) were treated with VKA, 74 patients (55.6%) were treated with NOACs, rivaroxaban 54 (73.0%), and dabigatran 20 (27.0%), respectively. Patients with bleeding had more often hypertension (75.9% vs. 40.8%, *p* < 0.001) and heart failure (27.1% vs.15.3%, *p* = 0.004). Amiodarone was more commonly used in patients with bleeding than those without bleeding (16.9% vs. 27.8%, *p* = 0.008). History of bleeding (19.5% vs. 8.0%, *p* < 0.001) and history of thrombosis (27.1% vs. 11.1%, *p* < 0.001) were more common in patients with bleeding. Gingival bleeding was more frequently reported than nasal bleeding and subcutaneous hemorrhage in all patients (49 cases (11.0%) vs.23 cases (5.1%) vs.16 cases (3.6%)) (Table 1).

### Factors influencing bleeding

**FIGURE 5:**
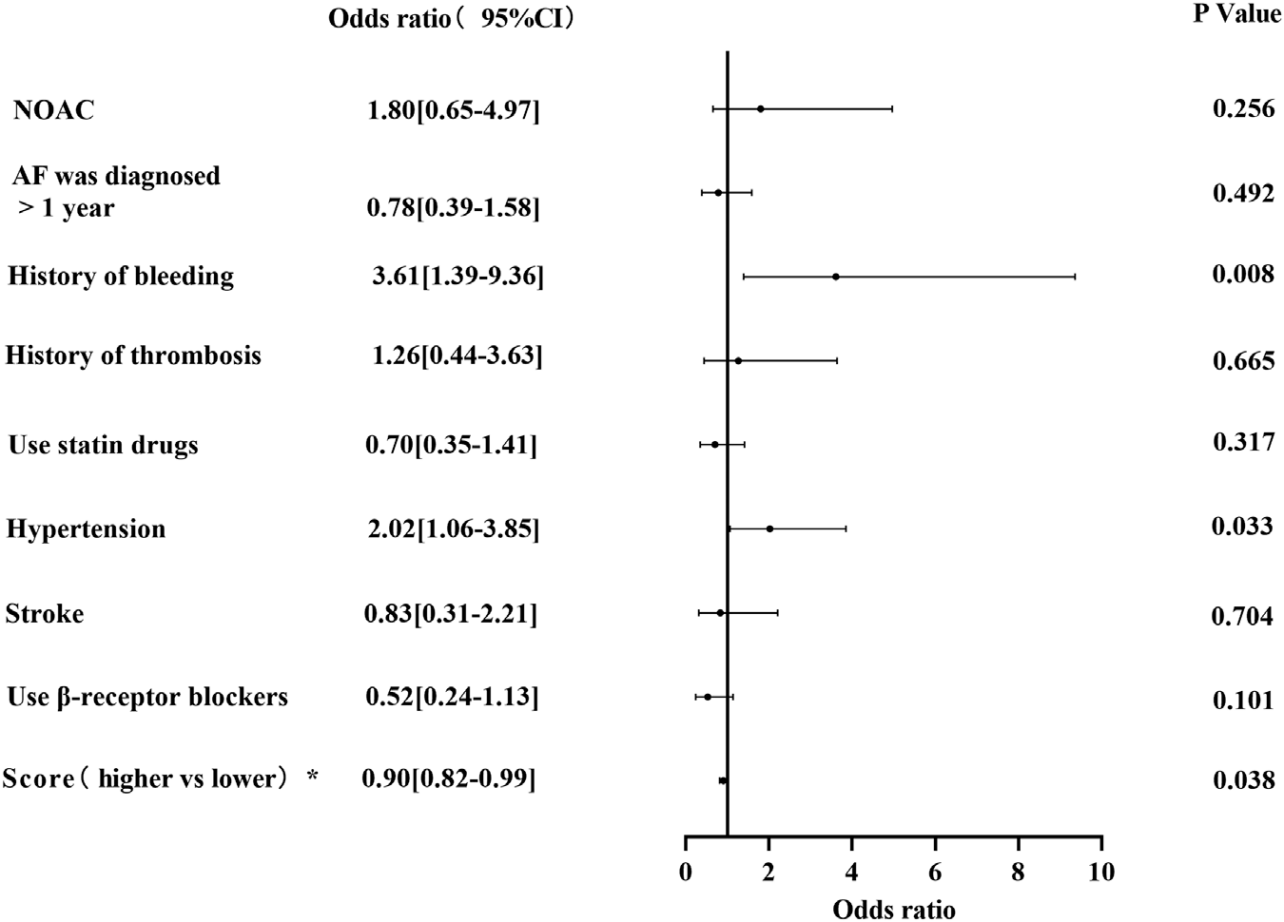
Factors associated with bleeding (*Score is a continuous variable).

In the multivariate-adjusted logistic regression model, the risk factors associated with bleeding were a history of bleeding (OR 3.61; 95%CI, 1.39–9.36; *p* = 0.008) and hypertension (OR 2.02; 95%CI, 1.06–3.85; *p* = 0.033). Meanwhile, the higher JAKQ score (OR 0.90; 95%CI, 0.82–0.99; *p* = 0.038) was a protective factor for bleeding (Figure 5).

## Discussion

In this multi-center trial, we validated the Chinese version of JAKQ, and used validated JAKQ to investigate the knowledge level of Chinese patients with AF about the disease and OAC, and to investigate the association between patients’ knowledge and bleeding. The main findings were: 1) The Chinese version of JAKQ was a reliable and effective tool for evaluating anticoagulant knowledge of AF; 2) Knowledge about AF and OAC in Chinese patients with AF was generally low; 3) Higher JAKQ scores were associated with secondary education or above, income more than 2000 yuan and history of AF for more than 1 year; 4) Bleeding in patients with AF was associated with lower JAKQ scores, hypertension, and history of bleeding.

In this study, JAKQ was systematically translated into Chinese for the first time, and it was used to evaluate the disease and OAC knowledge level of patients with AF at multiple centers in China. We translated the questionnaire into Chinese using a widely used method that meets the criteria in the Translation Guide (Wild et al., 2005)^.^ In the application of JAKQ, the questionnaire has acceptable internal consistency, good repeatability, and time stability, which proves that the Chinese version of JAKQ retains the reliability and validity of the original (Desteghe et al., 2016). The analysis shows that JAKQ is an effective tool for assessing the knowledge level about AF and OAC and can identify knowledge gaps in different aspects, addressing the most important issues in the management of AF and OAC.

The results of the knowledge assessment of Chinese patients with AF indicate that the median JAKQ score of patients with AF was 31.3%, which is much lower than the results of large population surveys (55.5%–62.5%) conducted by researchers in Belgium (Desteghe et al., 2016; Desteghe et al., 2019) and Poland (Janion-Sadowska et al., 2019). It is worth noting that the study population in this experiment came from 7 large Grade III A hospitals in cities, so the whole knowledge level of Chinese patients with AF may be even lower. One possible reason is that the overall average education level in China is lower than that in Europe (Patrinos and Angrist, 2018), and many primary care physicians know little about AF and anticoagulant treatment (Ye et al., 2020). Another possible reason is that the average consultation time of general practice in European countries is 10.7 min (Deveugele et al., 2002), while the median consultation time of Chinese general practice is only 2 min (Jin et al., 2015). It is interesting that the mean score increased again 2 months after education, compared to the 1-month score, and the knowledge scores of patients who did not participate in education did not increase significantly. This indicates that the increase in score has nothing to do with the increase in familiarity with the questionnaire, which highlights the importance of targeted education for patients by pharmacists. Meanwhile, repeated strengthening of targeted education may help patients have a deeper understanding of the disease and medication (Desteghe et al., 2019).

Several studies have shown that knowledge of AF and OAC is influenced by several socio-demographic factors, such as participants’ education level (Shen et al., 2020; Sedney et al., 2021), income (Tertulien et al., 2021), and duration of the disease (Desteghe et al., 2016). Similarly, our study found that patients with higher education, higher income, and longer disease duration had higher JAKQ scores. This can be explained by the fact that this group of people generally have a higher awareness of self-care and a stronger awareness of obtaining health information, which benefits their acceptance of disease-related knowledge. Meanwhile, patients with high education have a strong ability to accept new things and self-study (Shen et al., 2020; Sedney et al., 2021), while patients with low education and poor economic conditions are often unable to accurately grasp knowledge due to limited knowledge sources and relatively poor ability to accept and understand (Tertulien et al., 2021). Due to long-term treatment, patients with a long course of disease receive more guidance from the physician and have a relatively high knowledge score (Desteghe et al., 2016). Therefore, more attention should be paid to patients recently diagnosed with AF, and those with lower formal education and income. Pharmacists can spend more time educating such patients.

Physicians and patients often do not take anticoagulant therapy due to their fear of bleeding, so it is very important to evaluate the risk of bleeding in patients with AF. There have been studies discussing the management issues related to NOACs and special populations with increased risk of bleeding (Roberti et al., 2021). This study revealed some influential factors of bleeding, such as a history of bleeding and hypertension, which are similar to the results of previous studies (Ishii et al., 2017; Verdecchia et al., 2018; Rydberg et al., 2020). Therefore, more attention should be paid to the risk of bleeding in patients with AF who have a history of bleeding and hypertension during anticoagulant therapy. The selection of anticoagulant drugs for such patients should be more cautious.

Through the hypothesis that a lower JAKQ score was a risk factor for bleeding, we found that patients with bleeding during follow-up had poor knowledge levels of AF and OAC, and the validity of the instrument was proven. This was also confirmed by a cohort study using JAKQ (Konieczyńska et al., 2022), whose results showed that poor knowledge of AF and OAC in patients with AF was associated with an increased risk of adverse clinical outcomes, particularly clinically relevant bleeding, at a median follow-up of 42 months. It may be interesting to note that this study considered more serious forms of bleeding. The current findings suggest that by improving patients’ understanding of AF and OAC, it may be possible to reduce adverse clinical outcomes. It is also noteworthy that in our study 3 of the 4 questions for which the rate of correct responses were different between the patients with and without bleeding were broadly OAC-related, although the overall score reflected both AF and OAC-related knowledge. Equally important, few patients are aware that the combination of anticoagulants with widely used nonsteroidal anti-inflammatory drugs increases the risk of bleeding, especially gastrointestinal bleeding (Undas et al., 2020). Therefore, it is important to educate patients on the most appropriate painkillers to optimise the safety of their anticoagulant therapy. On the question “Do you need to go to the general practitioner every time you feel AF?” bleeding patients have a higher rate of the correct response. This may mean that bleeding patients are more likely to discuss their fears and concerns with their general practitioner, and receive more attention from their general practitioner (Metzgier-Gumiela et al., 2020).

It should be noted that although the knowledge about the disease in patients with AF is flawed, the best educational strategy for patients remains unclear (Heidbuchel et al., 2017; Hindricks et al., 2021). In the era of smart technology, applications on smartphones or tablets have been used to improve communication between patients with AF and healthcare professionals (Guo et al., 2017; Kotecha et al., 2018; Ayyaswami et al., 2019). Importantly, AF is associated with increasing age (Zhou and Hu, 2008; Hindricks et al., 2021). Yet, existing mobile health applications have design flaws that may limit usability by older adults (Li et al., 2021). Therefore, whether these technologies can achieve the best educational effect remains to be determined.

The study also has some limitations. First of all, all of our seven research centers are Grade III A hospitals in large cities, so our findings may not easily be extended to community service centers or secondary hospitals. Secondly, some bleeding and thrombotic events were self-reported by patients, which may have led to recall bias. In addition, our study was focused on the association between patients’ knowledge level and bleeding, and it remains to be established whether the scores on the JAKQ are related to patients’ adherence or satisfaction. Finally, suboptimal implementation of NOACs into clinical practice and some doctors not updating their understanding of anticoagulation therapy may have resulted in the low understanding of the OAC card among patients. This may be one of the reasons for the low score of Chinese patients with AF.

## Conclusion

In conclusion, the Chinese version of JAKQ shows good reliability and validity, indicating that it is a valuable tool for AF and oral anticoagulation (OAC) knowledge assessment. It can be used in clinical practice to guide educational activities and improve the effectiveness and safety of treatment. It was shown that Chinese patients with AF have insufficient knowledge about AF and OAC. Lower JAKQ scores are associated with bleeding, so targeted education is necessary. Targeted educational efforts should focus on patients recently diagnosed with AF and those with lower formal education and income.

## Data Availability

The original contributions presented in the study are included in the article/Supplementary Material, further inquiries can be directed to the corresponding author.

## Abbreviations

AF, atrial fibrillation; CI, confidence interval; INR, international normalized ratio; JAKQ, Jessa Atrial fibrillation Knowledge Questionnaire; OAC, oral anticoagulation; NOAC, non-vitamin K antagonist oral anticoagulants; VKA, vitamin K antagonists.
