# Supplementary material for: An instrument to measure atrial fibrillation knowledge in Chinese patients: validation of the Jessa Atrial fibrillation Knowledge Questionnaire
Source: Front Pharmacol. 2023 Jun 13;14:1148524. doi: 10.3389/fphar.2023.1148524 (PMC10321709; doi:10.3389/fphar.2023.1148524)
Supplement: Supplementary file 1 [file Table2.DOCX]

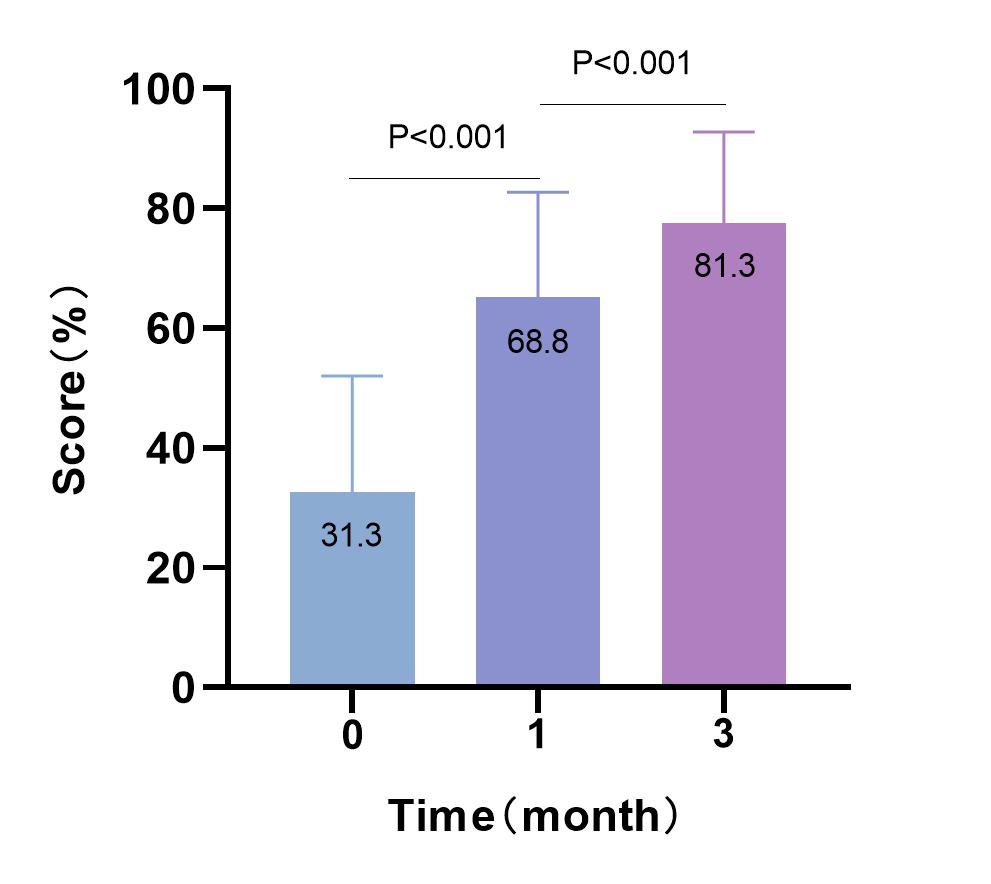


Figure 3 Effect of targeted education on the score of the Jessa AF Knowledge Questionnaire (JAKQ).

Validation JAKQ

Association of bleeding with score (n=447)

Validity

Intervention study (n=116)

Sensitivity

testing

Internal consistency

Cronbach’s alpha (n=447)

Test-retest reliability (n=135）

Reliability and repeatability

Figure 2 Overview of the different steps in the validation process of the Jessa Atrial fibrillation Knowledge Questionnaire.

AF: atrial fibrillation, and JAKQ: Jessa Atrial fibrillation Knowledge Questionnaire.

Chinese version

A1

Chinese version

A2

Chinese version

A12

English version

A3

JAKQ

**Backward**

**translation**

**Comparison**

**Consistent**

**Integration**

Figure 1 Translation process


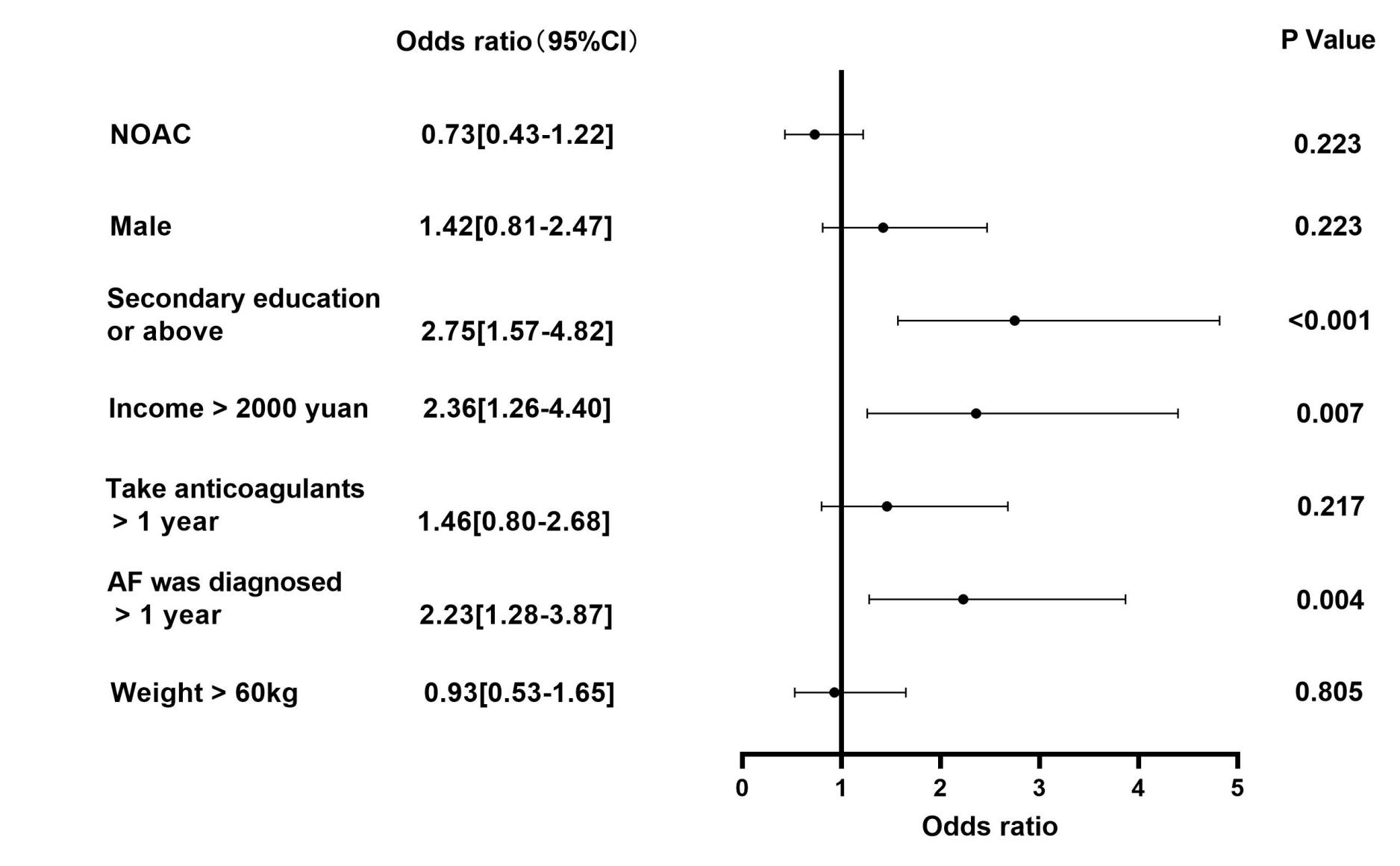


Figure 4 Factors associated with higher JAKQ score

A JAKQ score exceeding 50% was considered a high JAKQ score, otherwise it was considered a low score.


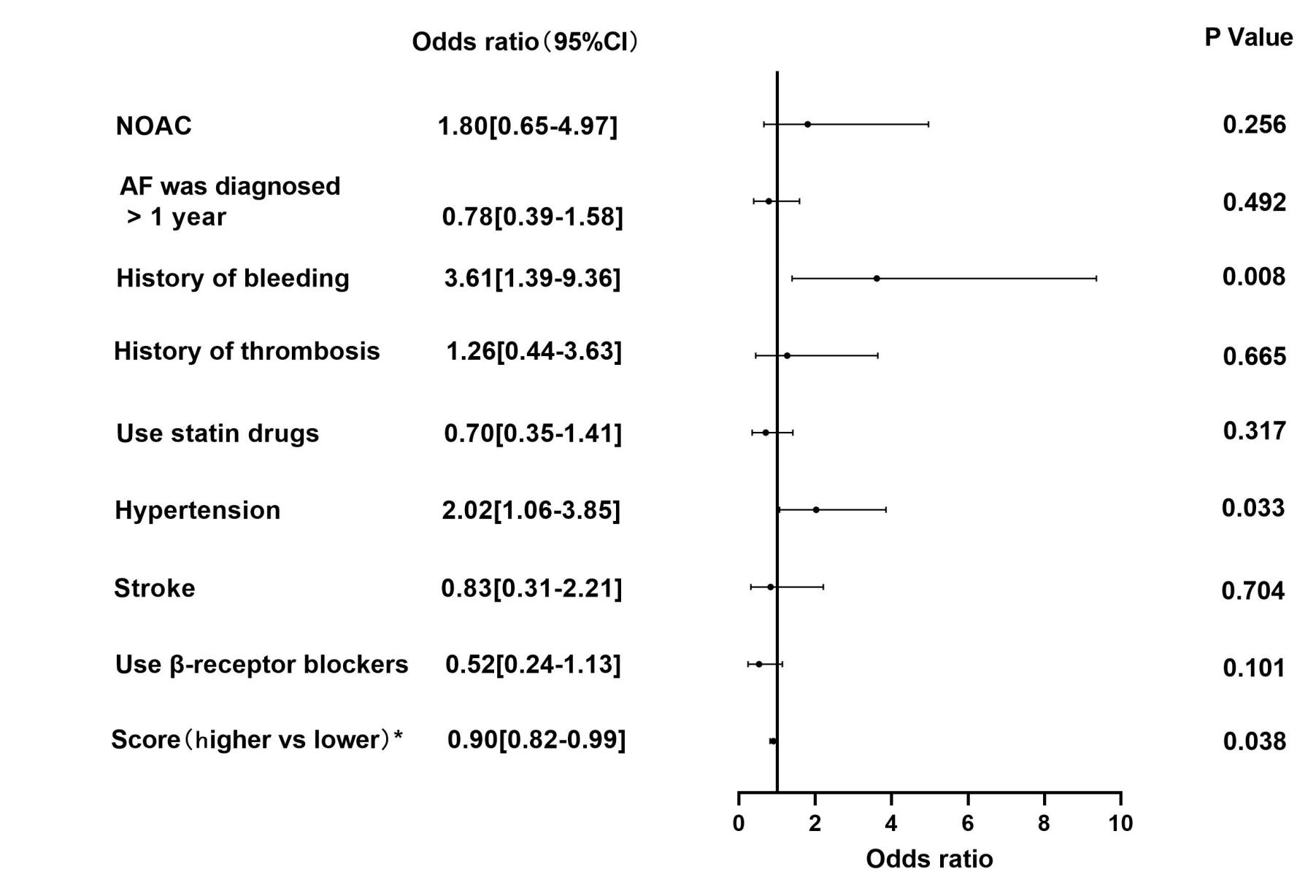


Figure 5 Factors associated with bleeding

*Score is a continuous variable


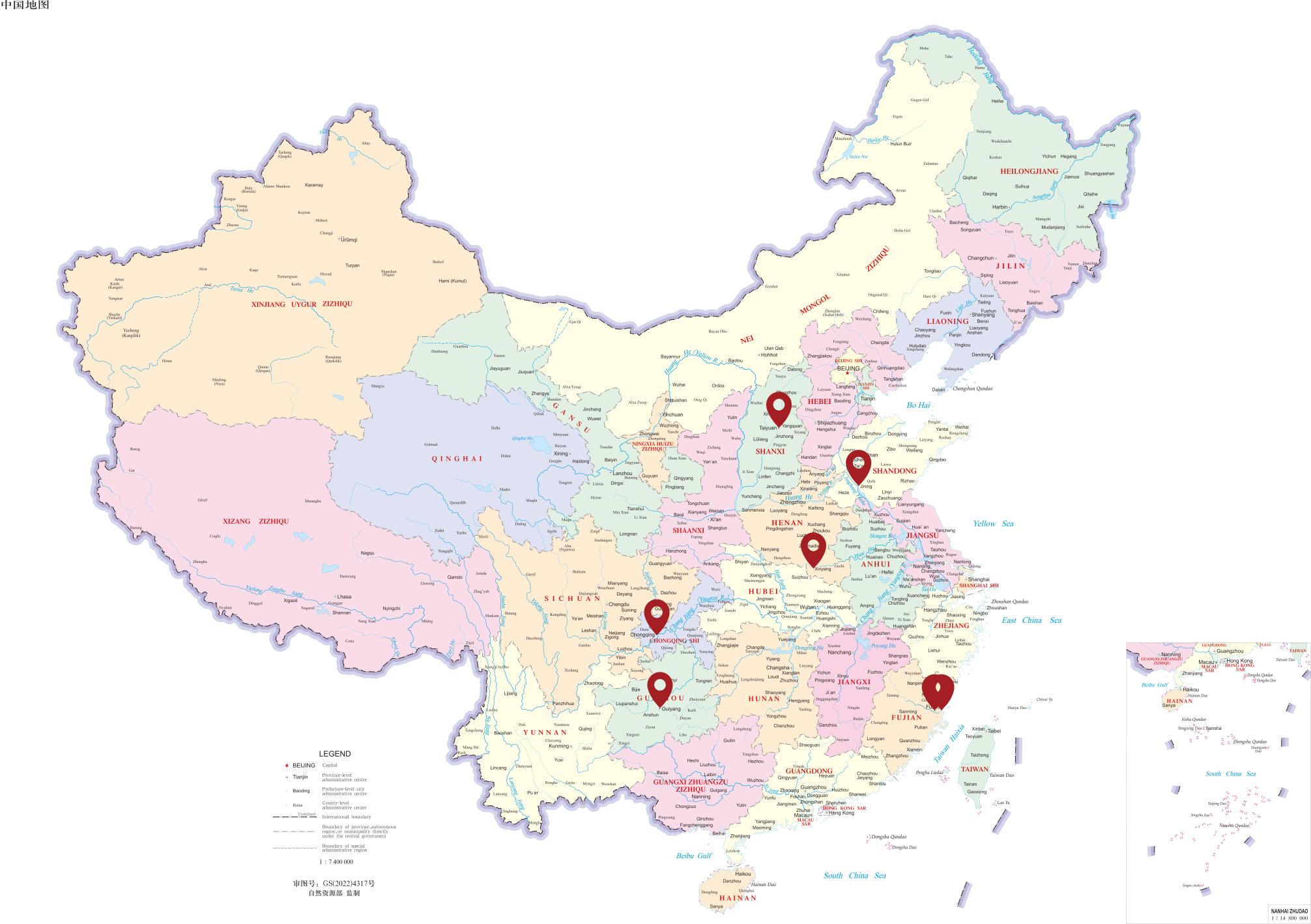


Supplementary Figure 1 Distribution map of each center
